# Macrocystic Adult Penile Lymphangioma: A Rare Presentation

**DOI:** 10.1155/2020/2608365

**Published:** 2020-08-11

**Authors:** Jaisukh Kalathia, Kaushal Patel, Santosh Agrawal

**Affiliations:** Department of Urology and Kidney Transplantation, Fortune Urology Clinic, India

## Abstract

Adult penile lymphangioma (APL) is a rare multifactorial vascular malformation. Usually, it is asymptomatic and located on the coronal sulcus or shaft of the penis. It is benign in nature with rare chances of recurrence. APLs can be approached via various modalities from simple “watch and wait” to electrofulguration, laser ablation, and surgical excision. We report a rare variant of lymphangioma of the penis inculcating the shaft being excised surgically with favorable outcome along with brief review of literature.

## 1. Introduction

Lymphangioma is a benign condition which may occur due to abnormal development of the lymphatic system. Lymphangiomas are usually found in the head and neck region with rare exception of genital involvement. There are only few reported cases of penile lymphangioma in the literature. The congenital variant become more apparent over time as reported by Handa et al. [1], whereas the acquired variant could occur as a result of lymphatic vessel occlusion due to infections such as frequent cellulitis [2]. These lymphatic malformations are further classified as microcystic (lymphangioma circumscriptum), cavernous lymphangiomas, macrocystic (cystic hygromas), and the acquired lymphangiomas (lymphangiecatsis) [3]. The signs and symptoms vary depending on the size and location of the mass. They can disfigure the affected area and may potentially disrupt the function if left untreated.

## 2. Case Report

A-24-year-old unmarried male patient presented with a mass over dorsum of the penis for the past 10 years. It was painless and gradually increasing in size. As per the history, there were no predisposing factors. On examination, the mass was approximately of size 3 cm × 2 cm lying on the dorsal aspect at the midshaft of the penis. It was nontender, mobile, and firm in consistency with absent inguinal lymph nodes. The ultrasonography of the penis showed cystic mass involving midshaft of the penis.

**Figure 1:**
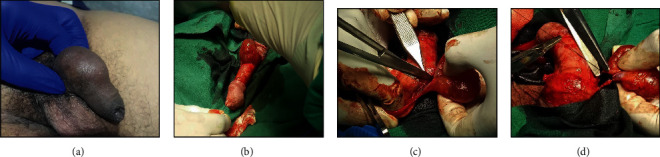
(a) Cyst involving shaft of penis. (b) Degloving of penis. (c) Dissection of cyst. (d) Ligation of the two feeding vessels.

**Figure 2:**
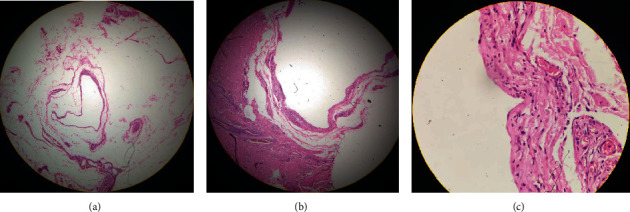
(a–c) Histopathological examination showing microcystic lymphatic malformation with irregular vascular channels and some containing slips of smooth muscle.

A circumferential subcoronal incision was given to deglove the penis. The plane was developed below the external Colles fascia remaining above the Buck's fascia to avoid inadvertent injury to neurovascular bundles. On proximal dissection, the cystic mass was found severely adhered to midshaft. The two large feeding vessels were found at the ventral aspect of the mass which were dissected and ligated so as to remove the mass intact (Figure 1). The postoperative period was uneventful, and the patient was discharged on postoperative day one. In follow-up, the patient had normal erection, and the histopathological examination confirmed benign vascular lesion suggestive of lymphangioma (Figure 2).

## 3. Discussion

Lymphangiomas or “lymphatic malformations” were first described by Redenbacher et al. in 1828 [4]. These lymphatic malformations are relatively rare and not connected with the lymphatic system. They are ubiquitous because of the universal distribution of the lymphatic system but are commonly found in the head and neck. Lymphangioma are usually diagnosed during infancy or childhood. But, only few acquired cases can manifest in early and late adulthood with incidence slightly higher in males. The congenital lymphangioma may result from failure of fetal lymph vessel to involute or to join with the central lymphatic system. However, multiple etiology including trauma, infections, radiotherapy, pregnancy, scleroderma, severe phimosis, or STDs may result in acquired lymphangioma [5].

So far, less than 50 cases have been reported since Ferris and Holmes first described penile lymphangioma in 1944 [6]. The lymphangiomas are classified based on depth and size of lymph vessels: (1) lymphangioma circumscriptum (most superficial) composed of small thin-walled lymphatics, (2) cavernous lymphangioma composed of large lymphatics, and (3) cystic lymphangioma or cystic hygromas (deepest) with major lymphatic dilatation [7].

Penile lymphangiomas are commonly located on the coronal sulcus or shaft of penis.

The presentation is asymptomatic in majority of patients but those symptomatic can have sexual dysfunction. Given the benign nature of penile lymphangioma and rare likelihood of recurrence, these can be managed conservatively in those asymptomatic. However, acquired large lymphangiomas causing disfigurement of the penis or for the cosmetic purpose surgical excision should be adopted.

Various treatment modalities can be offered to treat lymphangiomas from simple “wait and watch” to electrofulguration, laser ablation, and surgical excision with favorable prognosis in each. However, during surgical excision, staged excision may be required depending on size [8]. In our case, the large lymphangioma was surgically excised intact to prevent further disfigurement of the penis.

## 4. Conclusion

APL is a benign tumor of lymphatic system which can be excised surgically with favorable outcome to prevent long-term sequelae of being permanently disfigured.
